# The Challenge and Opportunity of NTRK Inhibitors in Non-Small Cell Lung Cancer

**DOI:** 10.3390/ijms23062916

**Published:** 2022-03-08

**Authors:** Haixia Qin, Manish R. Patel

**Affiliations:** Department of Hematology, Oncology and Bone Marrow Transplant, University of Minnesota, Minneapolis, MN 55455, USA; qin00095@umn.edu

**Keywords:** NTRK, non-small cell lung cancer, drug resistance

## Abstract

With the development of targeted therapy, non-small cell lung cancer (NSCLC) patients could have more treatment choices if target mutation presents. The neurotrophic tropomyosin receptor kinase (NTRK) has a low prevalence in NSCLC, roughly around 0.5%. FDA had approved two first generation NTRK inhibitors, larotrectinib and entrectinib. Both medications have excellent CNS penetration. This manuscript will review available data on targeting NTRK fusions in NSCLC and mechanisms of drug resistance.

## 1. Introduction

### 1.1. NTRK Structure and Physiological Function

The neurotrophic tropomyosin receptor kinase (NTRK) belongs to the tyrosine receptor kinase family, including NTRK1, NTRK2, and NTRK3, and codes for TRKA, TRKB, and TRKC, respectively.

NTRK1 was first identified as an oncogene in 1982 [1], and the cDNA of the NTRK1 proto-oncogene was isolated in 1989 [2]. In 1991, TRKA was found expressed in the nervous system. TRKA is a receptor for neurotrophin nerve growth factor (NGF) [3,4], and TRKB/TRKC were identified as members of the same family of receptors. These receptors can bind with different ligands: NGF for TRKA, brain-derived neurotrophic factor (BDNF) or neurotrophin 4 (NT-4) for TRKB, and neurotrophin 3 (NT-3) for TRKC [5]. All three NTRKs play a pivotal role in central nervous system development, including cell proliferation, migration, differentiation, and apoptosis.

**Figure 1:**
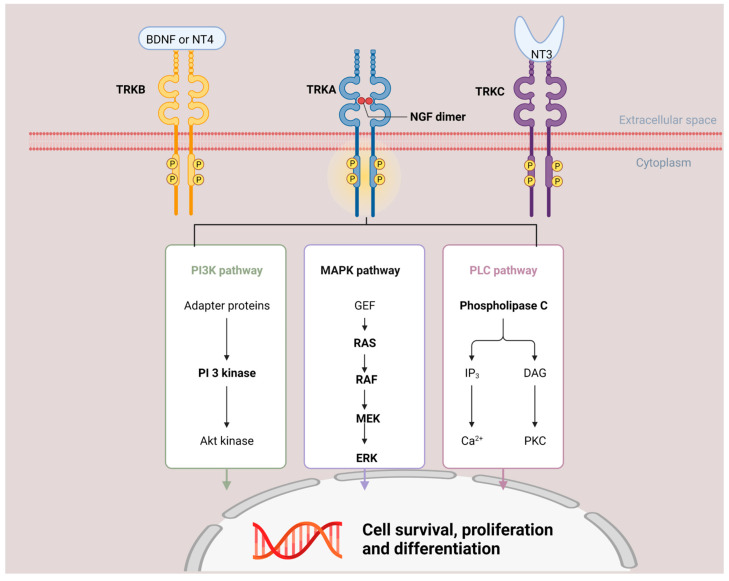
NTRK downstream signal pathway and function. NTRK has the typical structure of extracellular ligand binding domains, a transmembrane region, and intracellular kinase domain. When NTRK binds with its ligand, the downstream PI3K/MAPK/PLC pathway will be activated and cause cell survival, proliferation, and differentiation. BDNF—brain-derived neurotrophic factor; NT4—neurotrophin 4; NGF—neurotrophin nerve growth factor; NT3—neurotrophin 3; PI3K—Phosphoinositide 3-kinase; MAPK—Mitogen-activated protein kinase; PLC—Phospholipase C.

In 1997, Valent et al. first mapped NTRK1, NTRK2, and NTRK3 to human chromosomes 1q22, 9q22, and 15q25 by fluorescence in situ hybridization [6]. Subsequently, the structure and function pathway were studied extensively. Similar to other tyrosine receptor kinases, NTRK has the typical structure of extracellular ligand binding domains, a transmembrane region, and intracellular kinase domains. The downstream pathway for NTRK is listed in Figure 1. The Phospholipase C (PLC) pathway is physiologically activated downstream of NTRK activation, however, MAPK and PI3K pathways are implicated as well and perhaps more common with constitutive activation of NTRK as seen in malignancy expressing NTRK fusion [7]. The dominant signaling patterns are likely tissue and context dependent, and all of the above pathways can be activated by NTRK fusion proteins. 

**Figure 2:**
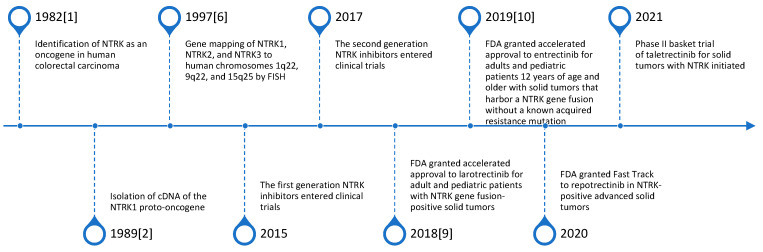
Timeline of NTRK genes and NTRK inhibitor therapy.

Though NTRK was identified as an oncogene in 1982, a long gap emerged prior to development of TRK inhibitors in 2015. Development of inhibitors of NTRK were somewhat delayed due to concerns about the normal function of TRK proteins in the central nervous system. Moreover, the crystal structure of the kinase domain of TRKA and TRKB was not resolved until 2012 [8]. This likely paved the way for development of TRK inhibitors that could be moved to clinical trials [8]. Thereafter, there has been very rapid development of activity in this area of study. Indeed, it was not until 2015 that the first NTRK inhibitors were tested in clinical trials. In just 6 years, two FDA-approved NTRK inhibitors and several 2nd generation drugs have reached clinical development [9,10]. The timeline of NTRK genes and NTRK inhibitor therapy is summarized in Figure 2.

### 1.2. Pathological Function of NTRK Fusions

Since NTRK was first identified as an oncogene in 1982 [1] in colon cancer, pathological NTRK fusions have been found in different cancer types. It is nearly universally found in mammary analogue secretory carcinoma [11], congenital/infantile fibrosarcoma, and congenital mesoblastic nephroma [12], in which the NTRK fusion often defines the diagnosis of these entities. NTRK fusions can be seen more commonly in thyroid and gastrointestinal stromal tumors (GIST) though the prevalence is highly variable amongst different studies due to variable patient sample sizes and methodologies employed [13,14]. However, it is much rarer in other common cancer types, at rates of <5%. For example, in one single center study from Memorial Sloan Kettering Cancer Center involving 33,997 cases, NTRK fusion was found in 17.7% of inflammatory myofibroblastic tumors, 5.08% of salivary gland carcinoma, and 2.28% of thyroid carcinomas. However, in other common tumor types such as lung, breast, colorectal, and pancreas, the rate was <1% [13].

While NTRK mutations, splice variants, and deletions can be seen in some tumor types, these alterations do not typically benefit from targeted therapy. Thus, this manuscript will focus on NTRK fusions as these are the ones associated with response to NTRK-targeted therapy. 

**Figure 3:**
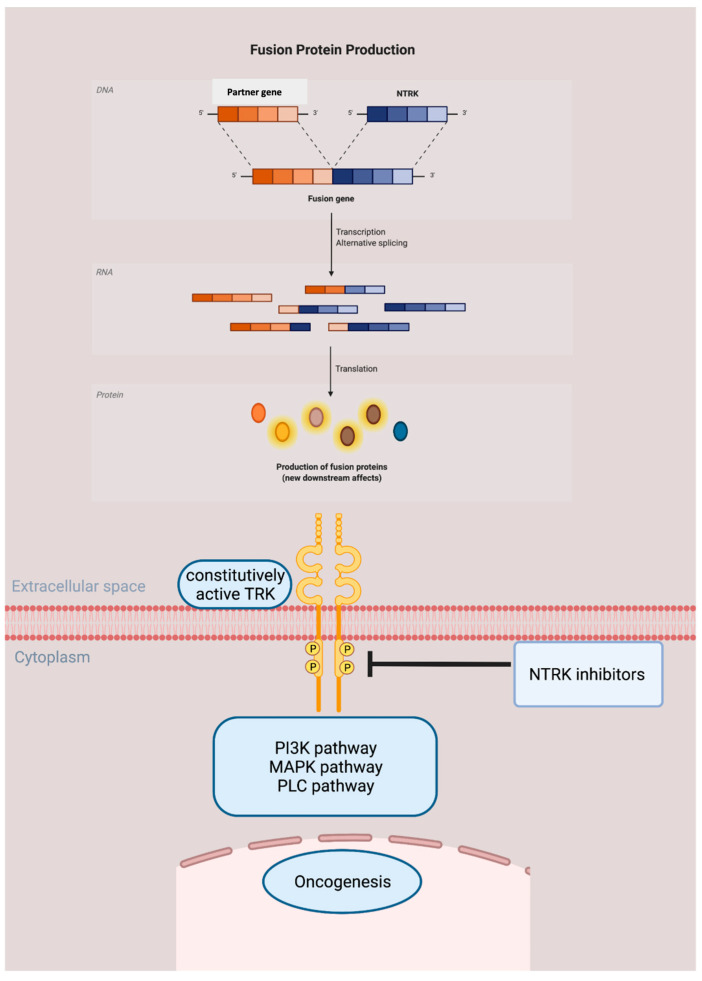
Oncogenesis pathway of NTRK fusion protein. The tyrosine kinase domain of NTRK fuses with the 5′ region of a partner gene, producing a chimeric protein. This fusion protein leads to constitutive activation of downstream pathways, resulting in ligand independent pathway signaling and subsequently cause oncogenesis. NTRK inhibitors are able to treat NTRK fusion malignancy by blocking the tyrosine kinase, hampering the signal pathway in both wide type and fusion protein producing tumor cells.

When the C terminal tyrosine kinase domain of NTRK fuses with the 5′ region of a partner gene, the chimeric protein leads to constitutive activation of downstream proteins, resulting in ligand independent pathway signaling [15], which will subsequently cause oncogenesis, as shown in Figure 3. 

So far, more than 80 different fusion partners to NTRK have been identified in various types of malignancy in both the adult and pediatric populations. Although fusion can occur in any NTRK gene, most of those identified occurred in NTRK1 and NTRK3. In mammary analogue secretory carcinoma, ETV6-NTRK3 translocation is found in 90% of cases. The detection of ETV6-NTRK3 differentiates this entity from other tumor types [7] and predicts response to NTRK inhibitors. Other common fusion partners include recurrent EML4-NTRK3 fusions in infantile fibrosarcoma [16], LMNA-NTRK1 fusion in a congenital infantile fibrosarcoma [17], TPM3-NTRK1 rearrangement in colorectal carcinoma, KCTD16-NTRK1 in ganglioglioma, and IRF2BP2-NTRK3 in papillary thyroid carcinoma [18]. The partner genes for lung cancer are summarized in Table 1.

## 2. NTRK Inhibitor

All currently available NTRK inhibitors are tropomyosin receptor kinase inhibitors (Trk) and some of them also have activity against ROS1 and ALK. NTRK inhibitors bind to Trk, thereby preventing ligand–Trk interaction and Trk activation, thus blocking the oncogenesis activity in cancer cells that overexpress NTRK fusion proteins, leading to both the induction of cellular apoptosis and the inhibition of cell growth in tumor cells. 

### 2.1. First Generation NTRK Inhibitors

The first generation NTRK inhibitors have been tested in clinical trials since 2015. Due to the rarity of NTRK fusions in any given tumor type, these trials have been predominantly basket trials. They have enrolled patients from a variety of tumor types expressing NTRK fusions. What follows will be a detailed discussion of the two US FDA-approved NTRK inhibitors, larotrectinib and entrectinib.

#### 2.1.1. Larotrectinib

Larotrectinib is the first pan-TRK selective inhibitor with a high selection for TRKA, TRKB, and TRKC. In 2018, Drilon et al. published the efficacy of larotrectinib in TRK fusion-positive cancers in both adults and children [9]. In this combined study, including three clinical trials LOXO-TRK-14001 (ClinicalTrials.gov identifier: NCT02122913), SCOUT (NCT02637687), and NAVIGATE (NCT02576431), a total of 55 patients (age from 4 months to 76 years) with 17 unique TRK fusion-positive tumor types, were enrolled and treated. In this study, Larotrectinib 100 mg twice daily (BID) was used for adults and children who had a body-surface area of at least 1 m^2^. For children who had a body-surface area of less than 1 m^2^, 100 mg per square meter BID was used. The overall response rate (ORR) was 75% (95% confidence interval (CI), 61–85) by blinded independent central review and 80% (95% CI, 67–90) according to investigator assessment. At a median follow up of 9.4 months, 86% of the patients with a response were continuing treatment or had undergone curative-intent surgery. The side effects were mostly gastrointestinal side effects, but approximately 30% of patients also developed dizziness. The vast majority of toxicity was Grade 1 and manageable. Based on these three multicenter, single-arm trials, the FDA granted accelerated approval to larotrectinib for adult and pediatric patients with NTRK gene fusion-positive solid tumors in November 2018. The updated efficacy and safety data with longer follow up were reported in the 2021 ASCO meeting. In the updated study, a total of 218 patients were treated with larotrectinib, and 206 were evaluable for efficacy. Among them, 44% of patients were positive for NTRK1, 3% positive for NTRK2, and 53% positive for NTRK3. A total of 21 different tumor types were treated: soft tissue sarcoma (STS) (46%; 20% infantile fibrosarcoma and 26% other STS), thyroid (13%), salivary gland (11%), lung (9%), and colorectal (5%). The age ranges from 0.1 to 84 years with a median age of 38.0 years. In total, 45% of patients had received two or more prior lines of systemic therapy, and 27% had no prior lines of systemic therapy. The ORR was 75% with 22% complete response (CR), 53% partial response (PR), 16% stable disease (SD), and 6% progressive disease (PD). The median progression free survival (PFS) was 35.4 months (95% CI 23.4–55.7) with a median follow up of 20.3 months. No new safety signals were observed in the safety analysis set, which included 53 patients on treatment for more than 24 months. Treatment-related adverse events (TRAEs) were mainly Grade 1–2, with 18% having Grade 3–4 TRAEs. Only 2% of patients discontinued due to TRAEs. Therefore, it is becoming clear that larotrectinib is a safe medication with powerful effects [24].

In the ASCO 2021 meeting, the data from clinical trials NCT02576431 and NCT02122913 focusing on lung cancer were also updated. All 20 patients were heavily pre-treated with a median of three systemic therapies (range 0–6) and a median age of 48.5 years (range 25.0–76.0 years old). A total of 19 patients had non-squamous non-small cell lung cancer, and one had neuroendocrine carcinoma. NTRK1 gene fusion was present in 80% of patients, and NTRK3 was present in 20% of patients. Patients were given larotrectinib 100 mg PO BID on a continuous 28-day schedule until disease progression, withdrawal, or unacceptable toxicity. Among 15 evaluable patients, ORR was 73% (95% CI 45–92) with 1 CR, 10 PR, 3 SD, and 1 PD. The median time to response was 1.8 months. Median overall survival was 40.7 months (95% CI 17.2 to not estimable) at a median follow up of 16.2 months. Adverse events were predominantly Grade 1–2. Two patients experienced Grade 3 events, including myalgia, hypersensitivity, and weight increase. No treatment was discontinued due to TRAEs [25]. Thus, this study supports the screening and use of NTRK targeted therapy in lung cancer patients with NTRK fusion.

#### 2.1.2. Entrectinib

In 2019, an integrated analysis of three phase 1 and phase 2 trials regarding entrectinib in patients with advanced or metastatic NTRK fusion-positive solid tumors suggested that entrectinib could induce durable and clinically meaningful responses [10]. There has been a total of four trials studying entrectinib, including STARTRK-1, STARTRK-2, ALKA-372-001 (all patients aged 18 years or older with metastatic or locally advanced NTRK fusion positive tumors), and STARTRK-NG (young adult and pediatric patients aged ≤21 years) [10]. A total of 54 patients with 10 different tumor types and 19 different histology types received at least one dose of entrectinib, regardless of tumor types or NTRK fusion partner. With a median follow-up time of 12.9 months, 7% had CR and 50% had PR. The median duration of response was 10 months (95% CI 7.1 to not estimable). While the majority of the toxicities were Grades 1 and 2, neurologic complications such as dizziness were seen in about 27% of patients. Weight gain was common and was the most common Grade 3 or 4 treatment-related adverse events observed (10% in the NTRK fusion-positive safety population and 5% in the overall safety-evaluable population). The most common serious treatment-related adverse events were nervous system disorders including dizziness and cognitive changes (three (4%) of 68 patients and 10 (3%) of 355 patients, respectively). No treatment-related deaths occurred. Based on these studies, on 15 August 2019, the FDA granted accelerated approval to entrectinib for adults and pediatric patients 12 years of age and older with solid tumors that harbor a NTRK gene fusion without a known acquired resistance mutation to NTRK inhibitors. The European Medicines Agency also approved Entrectinib in 2020. In 2021 the patient reported outcomes findings from STARTRK-2 suggest a favorable safety profile of entrectinib with minimal treatment burden [26].

### 2.2. The First Generation NTRK Inhibitors’ Central Nervous System (CNS) Activity

Because of the known important function of NTRK proteins in the CNS, larotrectinib was intentionally designed to limit CNS penetration. As a result, CNS activity was not categorized in a detailed way in studies using larotrectinib. CNS activity was sought in the entrectinib study, and 22/54 (22%) patients had CNS metastasis compared to <10% with larotrectinib. In the updated entrectinib trial, six (50%) had a PR per blinded independent central review, and four (33%) had stable disease [10]. Median time to CNS progression was 17 months (95% CI 14.3 to not estimable). In the initial larotrectinib trial, there were only nine patients with primary CNS tumors. In the extended study published in 2021, 19/218 (8.7%) patients had brain metastases at baseline, with 15 evaluable for efficacy. The ORR for patients with brain metastases was 73% (95% CI 45–92): 11 PR, 2 SD, and 2 PD [24]. Though it has not been carefully monitored in larotrectinib trials, it does appear that there is CNS activity. In the extended study of larotrectinib in lung cancer patients harboring NTRK fusion, 8 patients with baseline measurable and non-measurable CNS metastases were evaluable. The ORR was 63% with 5 PR, 2 SD, and 1 PD [25]. Although intracranial ORR was not carefully assessed in this trial, CNS progression was uncommon on larotrectinib. Furthermore, there are also multiple published case reports of CNS objective responses that have been observed on larotrectinib [27,28,29]. While the CNS activity is more robustly detailed for entrectinib, the available data suggest that both larotrectinib and entrectinib are active against CNS disease.

## 3. Second Generation NTRK Inhibitors

Similar to other TKIs, the durability of the first generation NTRK inhibitors is often limited by mutations that affect NTRK inhibitors’ binding interactions, including both primary and acquired resistance [29,30]. Thus, 2nd generation NTRK inhibitors were developed to overcome these resistance mutations. The second generation NTRK inhibitors have smaller molecular weight and compact macrocyclic structure compared to first generation NTRK inhibitors. This compact macrocyclic structure circumvents the steric hindrance that prevents first generation NTRK inhibitors to bind the ATP-binding site in solvent front, gatekeeper, and xDFG mutations. Thus, 2nd generation inhibitors can overcome these on-target resistance mechanisms.

The molecular weight of larotrectinib is 428.44 g·mol^−1^, entrectinib is 560.65 g·mol^−1^, repotrectinib is 355.37 g·mol^−1^, and selitrectinib is 380.43 g·mol^−1^. The compact macrocylic structure helps accommodate the drug resistance structure from mutations in distinct regions of the active site of the TRK kinase domain and possibly compound mutations with multiple mutations in an active site. The crystal structures of larotrectinib, entrectinib, selitrectinib, and repotrectinib were studied in cellular modules of TRKA/B/C fusions and resistant variants with a subtype evaluated in xenograft tumor models [30]. In this study, the potency against TRKA/B/C fusions is highest in repotrectinib (IC50 < 0.2 nmol/L), followed by selitrectinib (1.8–3.9 nmol/L), entrectinib (0.3–1.3 nmol/L), and larotrectinib (23.5–49.4 nmol/L). Compared to 2nd generation NTRK inhibitors, first generation NTRK inhibitors has reduced potency in an array of TRK mutations, including solvent front TRKA G595R, TRKB G639R, TRKC G623R, TRKC G623E; gatekeeper TRKA F589L, TRKB F623L, TRKC F617I; xDFG TRKA G667C, TRKB G709C, TRKC G696C; and compound mutation TRKA G595R and TRKA F589L. More data should be available with further clinical practice. Below, is a description of the 2nd generation TRK inhibitors and the available clinical data reported in the literature.

### 3.1. Repotrectinib

Repotrectinib is a smaller compound than other available ROS1, TRKA-C, and ALK inhibitors, which is designed to accommodate the bulky, positively charged arginine side chain in the solvent front without any steric clashes. These solvent-front substitutions include TRKA G595R, TRKB G639R, and TRKC G623R [31]. The smaller size of the compound also aids its excellent CNS penetration. In 2018, one phase I/II study showed that repotrectinib achieved responses in patients with ROS1 or NTRK3 fusion-positive cancers who had relapsed on earlier-generation TKIs (crizotinib and entrectinib, respectively) due to ROS1 or TRKC solvent-front substitution-mediated resistance [31]. For example, in the phase 1 study, a 44-year-old patient with metastatic mammary analogue secretory carcinoma and acquired ETV6–NTRK3 G623E mutation achieved PR after the failure of crizotinib and entrectinib. Repotrectinib dose was initially 40 mg once daily. It was gradually escalated to 160 mg BID as permitted by the protocol when the disease progressed and resulted in the reestablishment of disease control and remained on therapy for 17+ months with no dose-limiting toxicities. The side effects include mild Grade 1 ataxia, paresthesia, nausea, mild Grade 1 perioral numbness, and dysgeusia. Thus, the FDA has granted a Fast-Track designation to repotrectinib to treat patients with advanced solid tumors that have an NTRK gene fusion and have progressed on at least one prior line of chemotherapy and 1–2 prior TRK tyrosine kinase inhibitors.

### 3.2. Selitrectinib (LOXO-195)

Although the phase I/II study of selitrectinib is still ongoing (NCT03206931, NCT03215511), multiple case reports suggest that Selitrectinib could be a potent medication. In a 2021 report, one patient with mammary analogue secretory carcinoma of the parotid gland and ETV6-NTRK3 fusion was responding to entrectinib initially in the STARTRK-2 trial. He developed secondary resistance to entrectinib through NTRK3 G623R mutation and responded to selitrectinib [32]. In 2020, Hemming et al. reported a 47-year-old woman with metastatic unclassified sarcoma and TPM3-NTRK1 fusion initially achieved PR to larotrectinib (100 mg BID). After developing an acquired NTRK1 G595R solvent-front mutation, she was switched to selitrectinib (100 mg BID) and achieved PR at 3 months. The patient subsequently developed multiple sites of disease progression, which were surgically resected. Selitrectinib was resumed postoperatively, and she remained free of disease progression for more than 1 year [33].

### 3.3. Taletrectinib (DS-6051b/AB-106)

Taletrectinib (DS-6051b/AB-106) is a new highly selective ROS1/NTRK kinase inhibitor with potent preclinical activity against ROS1 G2032R solvent-front mutation, among others [34]. The first-in-human U.S. phase I results of taletrectinib were published in September 2020 [35]. In this study, a total of 46 adult patients with neuroendocrine tumors, tumor-induced pain, or tumors harboring ROS1/NTRK rearrangements were eligible. The most common treatment-related adverse events were nausea (47.8%), diarrhea (43.5%), and vomiting (32.6%). Pain score reductions were observed in the 800 mg once-daily dose cohort. The confirmed objective response rate was 33.3% among the six patients with RECIST-evaluable crizotinib-refractory ROS1+ NSCLC. In this study, a cabozantinib-sensitive ROS1 L2086F was identified as an acquired taletrectinib-resistance mutation. Furthermore, one patient with TPM3-NTRK1 differentiated thyroid cancer achieved PR of 27 months at data cutoff. It is important to note that this patient had not received a 1st generation NTRK inhibitor prior to enrollment on this study. To date there is no clinical data on the effect of taletrectinib on overcoming resistance to 1st generation NTRK inhibitors. Preclinical evidence suggests that taletrectinib can be efficacious in cells harboring NTRK resistance mutations at IC_50_ < 100 nM, however, the NTRK G667C mutation was resistant to taletrectenib with IC_50_ of >300 nM [34]. On 18 June 2021, the first patient was dosed in a Phase II basket trial of taletrectinib for solid tumors with NTRK fusion in China. Table 2 summarizes the activity of NTRK inhibitors in published clinical trials. 

## 4. Drug-Resistance Mechanism and Subsequent Treatment Strategy

As expected, there would be drug resistance to both 1st and 2nd generation NTRK inhibitors. NTRK drug resistance can be classified into on-target resistance, which means drug resistance mediated by mutation of the TRK kinase domain, and off-target resistance, which activates bypass or downstream pathways [29,30,31].

Based on the structure of NTRK, on-target resistance can occur in the TRK kinase domain via mutations in the solvent-front position (TRKAG595R, G667C, TRKBG639R, TRKCG632R), gatekeeper position (TRKAF589L), or kinase activation loop xDFG position (TRKAG667S or TRKCG696A) [37]. The above is not an exhaustive list, and the resistance mutations continue to expand as additional cases are reported. In general, on-target resistance mutations should be targeted with next generation NTRK inhibitors that can overcome these resistance mutations. As suggested above, in patients with acquired NTRK resistance mutations, repotrectinib has demonstrated in clinical studies an excellent response rate. 

If the mechanism of resistance to first generation therapy is due to off-target acquired resistance, then use of 2nd generation NTRK inhibitors is not likely to be effective. So far, most of the data has come from clinical case reports. Some drug-resistance mechanisms were identified in patients receiving the second generation NTRK inhibitors as sequential treatment, so data presented below will include drug resistance to both 1st and 2nd generation TRK inhibitors. Accumulated data suggest RAS/RAF/MEK/ERK pathways were involved. One patient with CTRC-NTRK1 fusion-positive pancreatic cancer developed resistance to larotrectinib and selitrectinib via an acquired BRAF V600E mutation along with a subclonal KRAS G12D mutation [38,39]. A xenograft developed from this patient’s tumor also demonstrated outgrowth of a BRAF V600E-positive subclone when treated with larotrectinib at the time of acquired resistance. Thus, downstream MAPK pathway activation was hypothesized as the TRK-independent bypass resistance mechanism. This patient subsequently was treated with combination RAF and MEK inhibition with a demonstration of tumor regression. Another patient with the LMNA-NTRK1 fusion-positive colorectal cancer who initially responded to larotrectinib developed on-target resistance due to NTRK1 G595R solvent front mutation. This patient subsequently responded well to selitrectinib. Biopsy of the liver metastases after progression on LOXO-195 showed a KRAS G12A substitution, and a new KRAS G12D mutation emerged upon further disease progression. Similarly, chronic treatment of one LMNA-NTRK1-positive, NTRK1 G595R-mutant CRC cell line (LMNA-NTRK1, NTRK1 G595R) with selitrectinib demonstrated KRAS G12D acquisition, supporting KRAS pathway activation in NTRK drug resistance. 

One patient with PLEKHA6-NTRK1 fusion-positive cholangiocarcinoma developed acquired resistance to entrectinib due to an acquired high-level focal amplification of MET with no new on-target resistance identified. Since MET amplification drove TRK-independent resistance, the patient did not respond to selitrectinib. She was treated with MET inhibitor, Crizotinib, and responded well. Interestingly, in this case, post-progression cfDNA demonstrated reappearance of focal MET amplification in addition to 13 emergent missense mutations in MET, several of which are known to impair Crizotinib binding. Therefore, multiple resistance mechanisms are possible, which is further supported by animal studies and cell line studies. For example, the TPR-NTRK1 fusion kinase expression in immortalized mouse pancreatic ductal epithelial or mouse lung epithelial cells promotes rapidly growing tumors in mice [40]. The combination of entrectinib plus the MEK1/2 inhibitor cobimetinib dramatically forestalls the onset of drug resistance in vivo. Thus, this study supports the rationale of combined inhibition of TRKA plus MEK1/2 in NTRK1-driven cancers. One patient with ETV6-NTRK3 fusion-positive pancreatic cancer acquired a MEK1 (MAP2K1) P124S mutation when the patient progressed on selitrectinib, preceded with treatment of multikinase TRK inhibitor PLX7486. MEK1 P124S mutation was reported as a weak oncogene, but it has been proposed to confer resistance to targeted therapy in BRAF V600E-mutant melanoma patients [41]. Another patient with TPR-NTRK1 fusion-positive pancreatic cancer developed resistance to entrectinib by acquiring NTRK1 G595R and developed another ERBB2 S310F mutation after a short response to selitrectinib [42]. An adolescent with treatment-refractory non-rhabdomyosarcoma soft tissue sarcomas, initially failed after chemotherapy, radiation therapy, and surgery. He was found to have DCTN1-NTRK1 gene fusion. Although he responded quickly to larotrectinib with improved symptoms and reduced masses, the response did not last long, and he did not respond to next-generation TRK inhibitor selitrectinib [36]. 

**Figure 4:**
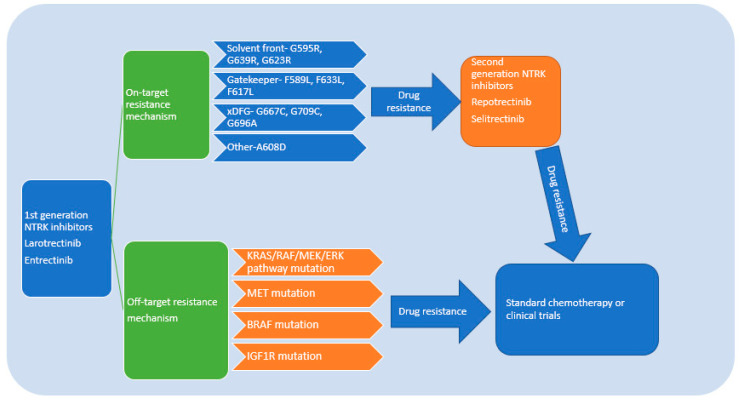
Drug-resistance mechanism of NTRK inhibitors and treatment algorithm. In NTRK fusion solid tumors, 1st generation NTRK inhibitors can achieve reasonable disease control. If there is only solitary site progression or oligoprogression, then 1st generation NTRK inhibitors can be continued while adding local therapy such as radiation or surgery. If drug resistance occurs to the 1st generation NTRK inhibitors, either through on-target or off-target mechanisms, a second generation NTRK inhibitor can be used to control the disease. If there are multiple targeted mutations, then combined therapy should be attempted to control the disease progression [43]. When the patient has no further targeted therapy after either 1st or 2nd generation NTRK inhibitors, then standard chemotherapy or clinical trials should be offered.

As is the case for other targeted therapies (EGFR, ALK, etc.), the optimal treatment of acquired resistance will likely depend on individual analysis of resistance mechanisms and targeting appropriately. Given more drug-resistance mechanisms identified, combination therapy by using several targeted therapies might be necessary for patients with multiple drug-resistance pathways. In the absence of other targetable mutations, standard chemotherapy and/or immunotherapy may be the only option. The above discussion probably means that serial next generation sequencing on tumor biopsy or cfDNA approaches will be necessary to treat patients adequately. Whether or not up-front combination therapies will be successful remains to be seen as it is not clear that there is a dominant form of resistance mechanism that can be broadly applied. Furthermore, the toxicity of such approaches will need to be carefully considered. The NTRK inhibitors’ drug-resistance mechanism and treatment algorithm are summarized in Figure 4.

## 5. Summary

The development of non-small cell lung cancer (NSCLC) targeted therapy has dramatically changed the outcome of metastatic lung cancer in the last decade. As with other driver mutations, NTRK fusion represents a small fraction of NSCLC that can benefit from targeted therapy. NTRK fusions, unlike other recurrent pathologic alterations, can be found in numerous different tumor types, and the response to NTRK inhibitors seems to be largely uniform regardless of the tumor type treated. Based on the dramatic improvement in outcome, we believe that NTRK fusion assay should be part of the standard assay for newly diagnosed patients with NSCLC. Since NTRK fusions are found rarely in many tumor types, one could consider it standard to assay for NTRK fusions in numerous tumor types as well. Though it is found only rarely, it will be missed if it is not assayed as a matter of routine. Importantly, we now know that resistance will develop, and it remains to be seen what the correct sequencing of 2nd generation NTRK inhibitors should be. Given the rarity of NTRK fusions, international consortia should be formed to analyze resistance mechanisms in a pooled fashion to develop larger datasets from real-world practice so that we can rapidly accumulate relevant data to make treatment decisions. Whether 2nd generation NTRK inhibitors appear to overcome resistance mutations, it will be interesting to see if 2nd generation NTRK inhibitors should only be applied after failure of 1st generation TKI or if they will supplant 1st generation NTRK inhibitors.

Although there are reports suggesting that high immunohistochemical expressions of TRKA, TRKB, and TRKC may predict for sensitivity to NTRK inhibitors [44], it remains to be seen if these findings are relevant to the clinical setting. In 2017, Ozono studied 99 cases of lung squamous carcinoma (SCC) samples and observed that the immunohistochemical expressions of TRKA, TRKB, and TRKC were observed in 33 cases (33%), 43 cases (43%), and 19 cases (19%), respectively [44]. Furthermore, high TRKB expression was significantly correlated with vascular invasion (*p* = 0.004) and lymph node metastasis (*p* < 0.001). In the same study, inhibition of TRKB, could suppress the invasion, migration, and proliferation activity of lung SCC cells. Squamous cell carcinomas are not typically responsive to targeted agents and there is a high unmet need in treatment refractory cases, thus, further study either in preclinical models or pilot clinical trials might be warranted.

## Figures and Tables

**Table 1:** NTRK fusion gene partners in lung cancer.

NTRK Gene	Fusion Partners	Cancer Type/References
NTRK1	TPM3	Adenocarcinoma [19]
	MPRIP	Lung cancer [20]
	CD74	Lung cancer [20]
	SQSTM1	Non-small cell lung cancer [21]
	IRF2BP2	Adenocarcinoma [22]
	TPR	Adenocarcinoma/neuroendocrine tumor [22]
	NCOR2	Adenocarcinoma [23]
	EPS15	Adenocarcinoma [13]
	TFG	Non-small cell lung cancer [22]
	F11R	Non-small cell lung cancer [22]
NTRK2	SQSTM1	Adenocarcinoma [19]
	STRN	Non-small cell lung cancer [22]
NTRK3	ETV6	Adenocarcinoma/squamous cell carcinoma [22]
	SQSTM1	Neuroendocrine tumor [22] Adenocarcinoma [13]
	RBPMS	Non-small cell lung cancer [22]
	EML4	Non-small cell lung cancer [22]

**Table 2:** NTRK inhibitors activity and side effects.

Medication	Larotrectinib [24]	Entrectinib [10]	Selitrectinib	Repotrectinib [36]	Taletrectinib
Generation	1	1	2	2	2
Target genes	TRKA/B/C	TRKA/B/C ROS1 ALK	TRKA/B/C	TRKA/B/C ROS1 ALK	TRKA/B/C ALK
Clinical trials	NCT02576431 NCT02122913 NCT02637687	ALKA-372-001 STARTRK-1 STARTRK-2	NCT03215511 NCT03206931	NCT03093116 (TRIDENT-1)	NCT04395677
Intracranial activity	Y	Y	Y	Y	N/A
Sample size (N)	206/218 eligible for efficacy evaluation	54	N/A	40	N/A
Cancer types	21	10			
Age (years)	38.0 (range 0.1–84.0)	58 (48–67)			
ORR	75% (95% CI 68–81)	31 (57%)	N/A	41–62%	
CR	45 (22%)	4 (7%)			
PR	109 (53%)	27 (50%)			
Stable	33 (16%)	9 (17%)			
PD	13 (6%)	4 (7%)			
Median duration of response (months)	49.3 (95% CI 27.3–NE)	10.4 (7.1-NE)			
Disease free progression time (months)	35.4 (95% CI 23.4–55.7) with a median follow up of 20.3 months	11.2 (8.0–14.9)			
Side effects	18% Grade 3–4 TRAEs.	10%-increased weight; 12%-anemia; 4%-nervous system disorders			
Brain metastasis result	N = 19 with 15 evaluable for efficacy: PR = 11 (73%); SD = 2 (13%); PD = 2 (13%)	N = 12 (22%): PR = 6 (50%); SD = 4 (33%)			
